# Breast-conserving surgery with intraoperative radiotherapy in recurrent breast cancer: the patient’s perspective

**DOI:** 10.1007/s12282-020-01114-y

**Published:** 2020-06-01

**Authors:** Constanze Elfgen, U. Güth, G. Gruber, S. Birrer, V. Bjelic-Radisic, M. Fleisch, C. J. Tausch

**Affiliations:** 1 Breast Centre Zurich, Seefeldstrasse 214, 8008 Zurich, Switzerland; 2 University of Witten-Herdecke, Witten, Germany; 3 Institute of Gynecology and Obstetrics, Universitätsklinikum Wuppertal, Wuppertal, Germany; 4 Institute of Radiooncology, Hirslanden Hospital Zurich, Zurich, Switzerland; 5 Faculty of Medicine, University of Zurich, Zurich, Switzerland

**Keywords:** Intraoperative radiotherapy, Recurrent breast cancer, Breast conserving surgery, Quality-of-life, Breast cancer, IORT

## Abstract

**Purpose:**

When ipsilateral breast-tumor recurrence (IBTR) following breast-conserving surgery (BCS) occurs, the cure of a potentially life-threatening disease is the main goal. If, however, this is diagnosed early, prognosis is still good and patient-reported outcomes become more important. Despite the fact that many patients would prefer a further BCS, international breast cancer guidelines still recommend mastectomy, mainly because previous radiation implies limited options. Our comparative study evaluates the long-term quality-of-life and outcome in patients with IBTR who received BCS plus intraoperative radiotherapy (IORT) versus mastectomy.

**Methods:**

Patients with IBTR were retrospectively divided into three groups according to the local treatment: group 1 (*n* = 26) was treated with BCS + IORT; group 2 (*n* = 35) received a standard mastectomy; group 3 (*n* = 52) had a mastectomy with subsequent reconstruction. Outcomes were analyzed after a mean follow-up of 5 years after IBTR. Quality-of-life was evaluated by the validated questionnaire BREAST-Q in 50 patients who fulfilled the inclusion criteria.

**Results:**

Quality-of-life scores varied within the groups, ranging from 51.4 to 91.3 (out of 100 points). We observed satisfactory scores in all items, with no statistical difference within the groups. Disease-free survival of all groups did not statistically differ, and overall mortality was very low (0.9%). The postinterventional complication rate was lower after BCS (19.2% versus 34.3% after mastectomy and 30.8% after mastectomy with reconstruction).

**Conclusion:**

For patients with previous surgery and radiation who demand a second BCS in the recurrent situation, this surgical technique can be offered in combination with IORT. Our long-term results imply oncological safety, lower complication rate, and good patient satisfaction.

## Background

Approximately, 3–20% of women with early breast cancer who received breast conserving surgery (BCS) and radiotherapy experience an ipsilateral breast-tumor recurrence (IBTR) during 10 years of follow-up [1, 2]. Previous treatment, in particular previous radiotherapy, is regarded as limiting the option of BCS, therefore, mastectomy is the recommended surgical treatment of IBTR after BCS [3, 4]. However, valid data from prospective studies that justify this procedure is not available. For some women, mastectomy is an accepted step and subjectively implies higher safety, although some patients would rather have a second BCS. Since many patients still have a good prognosis, quality-of-life (QoL) and patient’s satisfaction has become increasingly important in clinical practice [5]. In early breast cancer, patients report a better QoL and self-esteem after BCS than after mastectomy, even if breast reconstruction was performed [6]. This seems to be also true for the recurrent situation [7–9].

After whole-breast radiation, only partial breast irradiation is recommended and has to be indicated cautiously [10]. IBTR occurs mainly in the former tumor bed, due to remaining microscopic malignant cells in the surrounding tissue [11]. Based on that background, partial breast irradiation in the recurrent situation showed good results while minimizing radiation exposure to healthy breast tissue [9, 12].

Intraoperative radiotherapy (IORT) is defined as an application of single-dose irradiation delivered to the tumor bed during surgery. IORT provides a significantly improved cosmetic outcome and lower toxicity compared to whole-breast radiotherapy (WBRT) in early breast cancer [13]. However, data suggest a slightly higher IBTR rate in patients treated with single IORT compared to WBRT [14]. Weaknesses of the studies have resulted in controversy, and further long-term data are warranted [15]. To date, there are few studies of IORT in recurrent breast cancer, but they imply beneficial outcomes in selected patients [16, 17]. However, these studies did not consider QoL or patient satisfaction. Long-term QoL plays an important role in patients who receive the potentially traumatizing diagnosis and therapy of recurrent disease. This aspect has not been sufficiently studied until now. The primary aim of our study was to analyze the long-term QoL and satisfaction in patients with IBTR who received BCS + IORT. These data were compared to patients who received the still recommended standard therapy of IBTR, namely mastectomy with and without reconstruction. By doing so, we used the BREAST-Q, which is an established tool in measuring patient outcome at one point of time. It distinguishes physical outcome, breast satisfaction, sexual satisfaction, psychosocial well-being, and satisfaction with the surgeon. The BREAST-Q has been used in multiple studies to measure patient-reported outcomes, mostly in direct postoperative settings [18]. Outcomes and complication rates were retrospectively evaluated as a secondary endpoint in this study.

## Methods

Included in the study were patients from the Breast Center Zurich with IBTR of breast cancer, diagnosed between 2002 and 2018, who were primarily treated with BCS + RT (standard postoperative external beam radiotherapy) at early diagnosis. A total of 113 patients were retrospectively divided into three groups according to the IBTR treatment: group 1 (*n* = 26) received a secondary BCS [19] plus IORT. This technique delivers a high single boost dose to the former tumor bed in the intention to treat the area with the highest risk of local recurrence due to potential residual tumor cells. IORT was performed using high-energy electron (IntraBeam^®^) with an application surface dose of 20 Gy. The IORT applicator’s size was adapted to the tumor bed and ranged from 1.5 to 4.0 cm and the treatment time varied from 12–35 min. Group 2 (*n* = 35) received a mastectomy without reconstruction; group 3 (*n* = 52) had a mastectomy with subsequent reconstruction (autologous tissue or implant). Approximately half of the patients in all groups did not receive any axillary lymph node surgery in the recurrent situation, mainly because lymph nodes did not appear suspicious in the clinical imaging and marking of a sentinel lymph node failed due to previous surgery. Systemic treatment such as endocrine therapy and chemotherapy did not differ within the groups at early diagnosis nor at IBTR.

Quality of life was evaluated by the standardized and validated questionnaire BREAST-Q-BCT™ (BREAST-Q). The scales in BREAST-Q are scored numerically from 0 (worst) to 100 (best). Analysis was performed according to the questionnaire standards. Comparisons within the groups were performed for the five common scales of all postoperative modules: psychosocial well-being, sexual well-being, satisfaction with breasts, physical well-being, and satisfaction with surgeon [20]. Patients with metastatic disease, severe physical or mental restriction, or with insufficient language skills were excluded from the survey. More than 60% (*n* = 69) of all patients fulfilled the inclusion criteria for the questionnaire. Patient recruitment was done by personal contact or via phone call. If patients agreed to participate, they received an anonymized BREAST-Q for BCS, mastectomy, and mastectomy with subsequent breast reconstruction, respectively [20]. Study information, informed consent, and a prepaid envelope were attached. The contact rate was high (*n* = 66; 95.7%), as was the commitment to participate (*n* = 61; 88.4%), and the final answering rate (*n* = 52; 75.3%). Almost all returned questionnaires were completed for the clear majority of items, except the item “sexual well-being”; this item was sufficiently answered in 35 cases (67% out of 52). Two questionnaires had to be excluded from the analysis due to an insufficient rate of answered questions.

The descriptive analysis showed mean (and standard deviation) or median for continuous variables, and number and percentage for categorical variables. Locoregional and metastatic disease-free survival (DFS) after IBTR was illustrated by Kaplan–Meier plot according to Gebski et al*. *[21]. The F statistic was used to test for the joined significance of the group related indicator variable. All analyses were performed in the R programming language.

The study was conducted following the protocol approved by the Cantonal Ethics Committee of Zurich (BASEC-No. 2018-01191) and in accordance with Good Clinical Practice Guidelines. Patients granted a written consent to participate.

## Results

Histopathological tumor size, axillary lymph node status, tumor grading, and hormonal receptor status in early diagnosis as well as in IBTR did not differ within the groups. The time interval from initial diagnosis to IBTR was 10.5 years (mean; SD 7.8) and showed no statistical difference in all three study groups (Table 1). A longer follow-up after the treatment of IBTR was observed in the group of BCS + IORT (6.7 years) compared to the groups of mastectomy (4.8 years) and mastectomy with reconstruction (3.3 years). Patients with mastectomy and subsequent reconstruction were younger than in the other groups (55.4 years versus 64.6 and 69.0 years in BCS + IORT and mastectomy, respectively) (Table 1). In all groups, half of the patients did not received any re-surgical procedure in the axilla (44.2% to 53.8%).

**Table 1:** Patients’ and tumor characteristics in three treatment groups of local breast cancer recurrence

	Overall	BCS with IORT	Mastectomy	Mastectomy with reconstruction	*P* value
*N*	113	26	35	52	
Age at recurrence [years; mean (SD)]	61.8 (12.7)	64.6 (11.4)	69.0 (9.4)	55.4 (12.2)	< 0.001
Interval from early diagnosis to recurrence [years; mean (SD)]	10.5 (7.8)	11.6 (7.0)	12.3 (8.8)	8.6 (7.1)	0.07
Histopathological tumor size (*n*)					0.72
rpT1–2 rpT3–4 rpTx	91.2% (103) 5.3% (6) 3.5% (4)	96.2% (25) 3.8% (1) 0%	88.9% (31) 8.6% (3) 5.4% (1)	90.4% (47) 3.8% (2) 5.8% (3)	
Histopathological axillary lymph node (*n*)					0.01
rpN0 rpN1–2 rpNx	46.0% (52) 16.8% (19) 37.2% (42)	92.3% (24) 7.7% (2) 0%	22.9% (8) 25.8% (9) 51.4% (18)	34.5% (18) 17.3% (9) 46.2% (24)	
Tumor grading (*n*)					0.30
Higher differentiated (G1 and G2) Poorly differentiated (G3) Unknown (Gx)	44.2% (50) 40.7% (46) 15.0% (17)	61.5% (16) 34.6% (9) 3.8% (1)	0% (14) 45.7% (16) 14.2% (5)	38.5% (20) 40.4% (21) 21.2% (11)	
Hormonal receptors (*n*)					0.22
Positive (> 5%) Negative Unknown	67.3% (76) 28.3% (32) 4.4% (5)	80.8% (21) 19.2% (5) 0%	65.7% (23) 22.9% (8) 11.4% (4)	61.5% (32) 36.5% (19) 1.9% (1)	
Adjuvant chemotherapy (*n*)					0.09
Yes No Unknown	25.7% (29) 70.8% (80) 3.5% (4)	11.5% (3) 88.5% (23) 0%	25.7% (9) 71.4% (25) 2.9% (1)	30.8% (16) 61.5% (32) 7.7% (4)	
Adjuvant endocrine therapy (*n*)					0.08
Yes No Unknown	60.2% (68) 36.3% (41) 3.5% (4)	76.9% (20) 23.1% (6) 0%	62.9% (22) 31.4% (11) 5.7% (2)	50.0% (26) 46.2% (24) 3.8% (2)	
Axillary surgery					0.55
No axillary surgery Sentinel nodectomy Axillary dissection	48.7% (55) 38.1% (43) 13.3% (15)	53.8% (14) 42.3% (11) 3.6% (1)	51.4% (18) 34.3% (12) 14.3% (5)	44.2% (23) 38.5% (20) 17.3% (9)	

The return rate of the BREAST-Q was slightly higher in patients with BCS + IORT without statistical difference. Physical and psychosocial well-being was slightly higher scored in the group of BCS + IORT (Scores 77.1/80.8 versus 74.6/77.6 and 67.8/79.8 in mastectomy group and mastectomy with reconstruction group, respectively). The item scores of local satisfaction with breast/chest wall trended higher in the group of mastectomy with subsequent reconstruction (Score 70.4 versus 55.5 in BCS + IORT and 66.0 in mastectomy group). Satisfaction with the surgeon was also higher scored in this group (91.3 points versus 86.3 in BCS + IORT and 84.2 in mastectomy group). Sexual well-being showed best results in the group of mastectomy (Table 2). Despite these trends, there were no clinically important differences of questionnaire scores within the groups. Higher satisfaction scores in most items were related to higher age (> 75 years) in every group, but even taking this into consideration there was still no evidence of significant differences.

**Table 2:** Patient-reported outcome by BREAST-Q scores in five items

	Overall	BCS with IORT	Mastectomy	Mastectomy with reconstruction	*p* value
*N*	50	19	13	18	
Local satisfaction with breast/chest wall Mean (SD)	63.6 (22)	55.5 (19)	66.0 (19)	70.4 (25)	0.10
Psychosocial well-being Mean (SD)	79.6 (19)	80.8 (19)	77.6 (17)	79.8 (22)	0.90
Sexual well-being Mean (SD)	60.4 (27)	53 (28)	73.9 (25)	51.4 (25)	0.08
Physical well-being Mean (SD)	73.0 (18)	77.1 (16)	74.6 (21)	67.8 (17)	0.26
Satisfaction with surgeon Mean (SD)	87.6 (19)	86.3 (19)	84.2 (21)	91.3 (17)	0.55

**Fig. 1:**
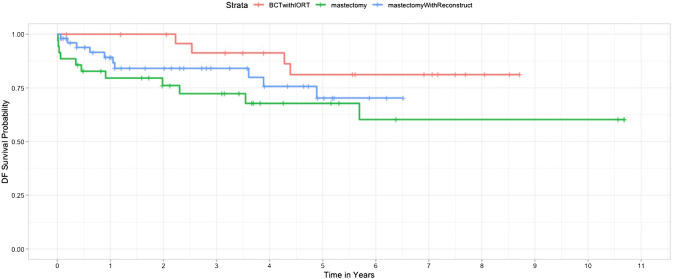
Locoregional and metastatic disease-free survival probability of patients with IBTR of breast cancer

Only one cancer-related death was observed during the follow-up period, which was in the reconstruction group. DFS did not differ significantly across all groups, but outcomes trended better in patients with BCS (Fig. 1). Postinterventional complications such as hematoma requiring intervention, wound infection, or chronic scar pain were lower after BCS + IORT (19.2% versus 34.3% after mastectomy and 30.8% after mastectomy with reconstruction) (Table 3).

**Table 3:** Postinterventional complications in the three treatment groups of local breast cancer recurrence (multiple answers possible)

	Overall	BCS with IORT	Mastectomy	Mastectomy with reconstruction	*p* value
*N*	113	26	35	52	
Postinterventional complications (*n*)	29.2% (33)	19.2% (5)	34.3% (12)	30.8% (16)	0.05
Hematoma Seroma	6.2% (7) 13.3% (15)	3.8% (1) 0	5.7% (2) 20% (7)	9.6% (5) 13.5% (7)	
Wound infection	8.0% (9)	0	8.6% (3)	11.5% (6)	
Chronic scar pain	2.7% (3)	11.5% (3)	0	0	
Lymph edema	2.7% (3)	3.8% (1)	2.9% (1)	1.9% (1)	

## Discussion

Outcomes after second BCS and partial breast re-irradiation is a very current topic in the literature [22, 23]. Recently, a study by Arthur et al. demonstrates the long-term safety of this procedure, emphasizing the results of the GEC-ESTRO trial [24, 25]. Since there are comparatively few long-term QoL studies in IBTR, we focused particularly on this item in our study. To the best of our knowledge, this is the first study in IBTR that compares the treatment options of BCS + IORT to mastectomy and mastectomy with reconstruction and provides the long-term outcomes of the three groups.

Overall, QoL questionnaires had good results (satisfaction scores from 60.4 to 87.6 out of 100). We observed no significant differences in QoL and patient’s satisfaction after BCS + IORT compared to the other groups. Taking previous studies into account, it was surprising that the results showed similar QoL scores after mastectomy without reconstruction [26, 27]. Patients in this study group were older, which can reflect patients’ and doctors’ preference to avoid possible risks and a prolonged surgery for reconstruction, and a more pragmatic attitude towards the treatment options. In elderly patients, BCS might have not the same relevance or preference as in younger patients. We observed a higher satisfaction in older patients for all groups. This could be related to a more positive attitude towards physical and mental health and medical treatment in older patients [28]. It may also show a stronger influence of desire for social agreeability or conformity when older patients participate in a survey. In all three groups of BCS + IORT, mastectomy, and mastectomy with reconstruction, we found no significant differences in the aspects of psychosocial, sexual, and physical well-being, or in local body satisfaction and in satisfaction with the surgeon. Our results cannot confirm an earlier study by Jendrian et al., which showed better QoL results after a second BCS in the recurrent situation; however not all patients included in this study received a partial re-irradiation [26].

Our entire study group showed an excellent overall survival, with just one cancer-related death (0.9%). DFS did not differ significantly in all groups, but outcome trended to be better in patients with BCS + IORT, implying the oncological safety of this procedure. This is also supported by the fact that median follow-up was longer in the BCS + IORT group than in the other groups. The secondary endpoint was adverse events such as prolonged wound healing, excessive seroma or hematoma, or necrosis and was lower in BCS + IORT compared to the mastectomy groups. These results are concordant to a low complication rate of IORT in earlier publications [13, 29].

Our study has some limitations and strengths which should be highlighted. An important point of all surgical comparison studies is the selection bias, which is also true in this study: women who choose BCS might have different attitudes and characteristics than women who choose mastectomy with or without subsequent breast reconstruction [30]. The follow-up time was longer in the group of BCS + IORT and reflects a general trend towards higher rates of mastectomy (with or without reconstruction) in the last few years [31]. Moreover, surgeons’ recommendations are also influenced by factors such as preoperative breast aesthetics, personal experience, and the availability of resources. On the other hand, the national health insurance system in Switzerland allows access to all surgical and conservative treatment options for all patients. Socio-economic confounders are, therefore, supposed to be much lower in the decision-making process than in other countries. The patients who received a second BCS + IORT in the recurrent situation were highly selected and desired breast preservation. However, tumor characteristics did not differ from the groups treated with mastectomy. A randomized trial would be necessary to exclude most confounders; but withdrawing the patient’s possibility to choose a surgical method in this context is ethically questionable and, therefore, impracticable [30]. For patients included in this study, treatment recommendation followed an interdisciplinary tumor board consent and an individualized decision-making process between patient and physician.

Recent studies have evidence to suggest oncological safety of secondary BCS and partial breast re-irradiation in the recurrence situation [10, 32]. However, there exist very few studies about IORT in IBTR of breast cancer and they have clear weaknesses, such as short follow-up time, lack of a control group, and very low patient numbers [16, 17, 33]. Our study includes data of 26 patients with IBTR treated with BCS + IORT, which clearly is a higher number than in other studies. However, because of the retrospective character of our analysis, outcome data have to be interpreted with caution. We first compared the treatment methods of BCS + IORT, mastectomy, and mastectomy with subsequent reconstruction regarding to long-term clinical outcome and quality of life in patients with breast cancer recurrence. For patients demanding a second BCS in recurrent situation, our results show equivalent outcomes of this treatment option in combination with IORT.

## Data Availability

The datasets used and analyzed during this study are available from the corresponding author on reasonable request.
